# Widespread continental mtDNA lineages prevail in the bumblebee fauna of Iceland

**DOI:** 10.3897/zookeys.774.26466

**Published:** 2018-07-16

**Authors:** Grigory S. Potapov, Alexander V. Kondakov, Yulia S. Kolosova, Alena A. Tomilova, Boris Yu. Filippov, Mikhail Yu. Gofarov, Ivan N. Bolotov

**Affiliations:** 1 Northern (Arctic) Federal University, Arkhangelsk 163002, Russian Federation; 2 Federal Center for Integrated Arctic Research of the Russian Academy of Sciences, Arkhangelsk 163000, Russian Federation

**Keywords:** Dispersal, Hymenoptera, invertebrate introduction, island biogeography, North Atlantic islands

## Abstract

Origins of the fauna in Iceland is controversial, although the majority of modern research supports the postglacial colonization of this island by terrestrial invertebrates rather than their long-term survival in glacial refugia. In this study, we use three bumblebee species as a model to test the hypothesis regarding possible cryptic refugia in Iceland and to evaluate a putative origin of recently introduced taxa. *Bombus jonellus* is thought to be a possible native Icelandic lineage, whereas *B. lucorum* and *B. hortorum* were evidently introduced in the second half of the 20^th^ century. These phylogeographic analyses reveal that the Icelandic *Bombus jonellus* shares two COI lineages, one of which also occurs in populations on the British Isles and in mainland Europe, but a second lineage (BJ-02) has not been recorded anywhere. These results indicate that this species may have colonized Iceland two times and that the lineage BJ-02 may reflect a more ancient Late Pleistocene or Early Holocene founder event (e.g., from the British Isles). The Icelandic populations of both *Bombus lucorum* and *B. hortorum* share the COI lineages that were recorded as widespread throughout Eurasia, from the European countries across Russia to China and Japan. The findings presented here highlight that the bumblebee fauna of Iceland comprises mainly widespread ubiquitous lineages that arrived via natural or human-mediated dispersal events from the British Isles or the mainland.

## Introduction

Iceland is a large North Atlantic island, the fauna of which is mostly of Palaearctic origin, with very few lineages that arrived from the Nearctic Region (Gíslason 2005, Gíslason et al. 2015, Novichkova et al. 2014, Pálsson et al. 2016, Bolotov et al. 2017). The majority of recent phylogeographic research supports the *tabula rasa* hypothesis, which suggests the allochthonous origin of freshwater and terrestrial fauna in Iceland since the last glaciation (Pálsson et al. 2016, Bolotov et al. 2017). However, the possibility of the long-term survival of several cold-adapted lineages on this island could not be excluded, at least for inhabitants of specific environments, e.g., groundwater (Kornobis et al. 2010).

Bumblebees (Hymenoptera: Apidae: *Bombus* spp.) are an appropriate model for biogeographic reconstructions because these insects are associated with flowering plants and are poorly equipped for dispersal across large water barriers (Bolotov et al. 2013, Potapov et al. 2017). The faunistic research of bumblebees in Iceland has a long history (Prŷs-Jones et al. 1981, 2016, Kristjánsson 2013, Kratochwil 2016). In summary, the fauna of Iceland comprises seven species, but only the *Bombus jonellus* (Kirby, 1802) is thought to be a native inhabitant of this island (Prŷs-Jones et al. 1981, 2016, Kratochwil 2016). This species appears to be at risk of decline due to the spread of invasive plant species such as Nootka lupine (*Lupinus nootkatensis*) and cow parsley (*Anthriscus sylvestris*) (Willow 2017). However, Prŷs-Jones et al. (1981, 2016) have suggested that it probably originated with a historical founder event via the arrival of hibernating queens on ships carrying Irish monks (8^th^–9^th^ centuries) or Vikings (9^th^–10^th^ centuries), or even later. *Bombus lucorum* (Linnaeus, 1761) and *B. hortorum* (Linnaeus, 1761) appear to have arrived in Iceland in the second half of the 20^th^ century, whereas *B. hypnorum* (Linnaeus, 1758), *B. pascuorum* (Scopoli, 1763), and *B. pratorum* (Linnaeus, 1761) appeared at the beginning of the 21^st^ century. Finally, *B. terrestris* (Linnaeus, 1758) is actively utilized as a pollinator in greenhouses and may have become naturalized in the country (Prŷs-Jones et al. 2016, Kratochwil 2016).

In spite of the fact that there have been multiple colonizations of Iceland by bumblebees, the origin of certain lineages has not been studied using a molecular approach, and only a single barcode sequence of *Bombus lucorum* from Iceland is currently available (Williams et al. 2012, Prŷs-Jones et al. 2016). Our objective herein is to test the hypothesis regarding possible cryptic refugia in Iceland and to evaluate a putative origin of artificially introduced taxa based on molecular sequence data inferred from three bumblebee species, i.e. *Bombus jonellus* (putative native lineage), *B. lucorum*, and *B. hortorum* (recently introduced species).

## Materials and methods

### Data sampling

**Figure 1.**
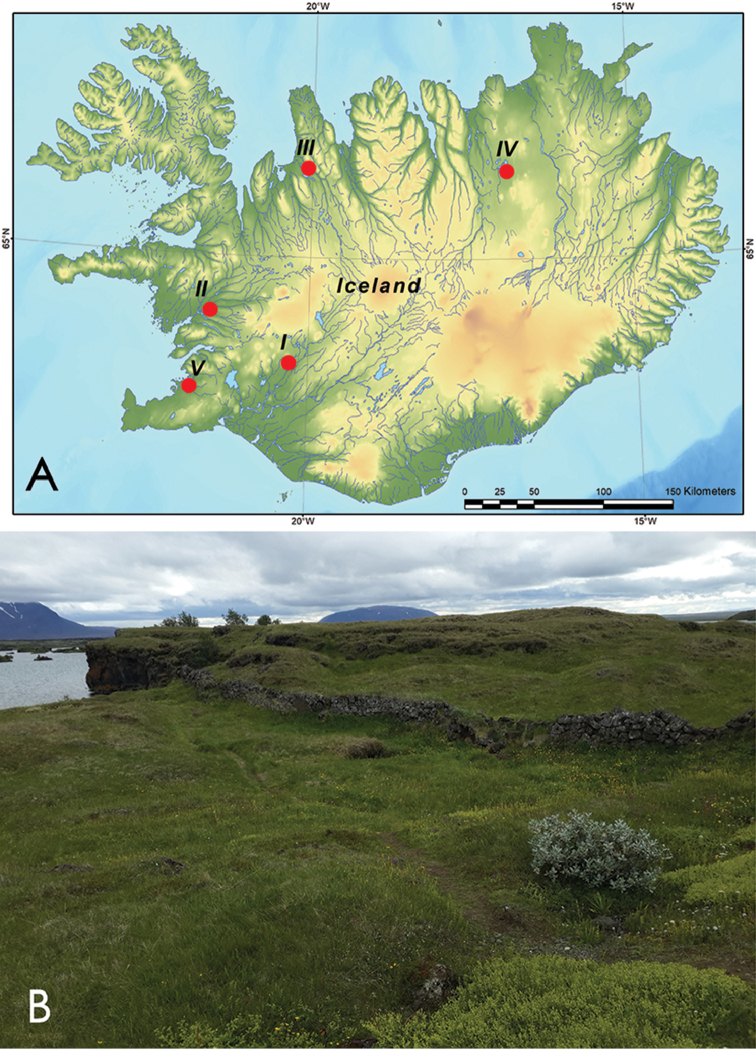
Map of study sites and typical habitat of *Bombus jonellus* in Iceland. **A** Map of study sites (see Table 1 for details). Red circles indicate sampling locations. **B** Shore of Mývatn Lake, a site with sympatric occurrences of the two lineages of *Bombus jonellus* (BJ-01 and BJ-02) in mountain herb-dwarf shrub tundra assemblages. Photograph by Mikhail Yu. Gofarov.

The bumblebee samples were collected with an entomological net (Ivan N. Bolotov leg.) in western and northern Iceland (Fig. 1). In summary, 64 individuals of three species were collected from five localities (Table 1). Specimens were deposited at the Russian Museum of the Biodiversity Hotspots (RMBH) of the Federal Center for Integrated Arctic Research of the Russian Academy of Sciences (Arkhangelsk, Russian Federation). Bumblebee species were identified following Løken (1973) and Rasmont and Terzo (2010). The species names are given in accordance with Williams (2018).

**Table 1.** Collecting localities and samples of bumblebees in Iceland.

Code	Localities	Coordinates	Date	Habitats	Species and samples
I	Geysir	64°18'50.9"N, 20°17'58.7"W	12.VII.2013	Mountain herb-dwarf shrub tundra assemblages on lava fields and geyserite	*Bombus lucorum* (2☿)
II	Flókadalsá River	64°37'4.2"N, 21°30'9.4"W	16.VII.2013	Roadside, Nootka lupine assemblages	*B. lucorum* (4☿); *B. jonellus* (2☿)
III	Blanda River	65°34'52.7"N, 20°2'59.7"W	17.VII.2013	Herb meadows	*B. lucorum* (19☿); *B. jonellus* (1☿, 7♂)
IV	Mývatn Lake	65°34'12.4"N, 16°57'12.7"W	17.VII.2013	Mountain herb-dwarf shrub tundra assemblages	*B. lucorum* (2☿); *B. jonellus* (11☿, 1♂)
V	Reykjavík	64°7'44.7"N, 21°47'12.6"W	18.VII.2013	Herb meadows	*B. lucorum* (2☿); *B. jonellus* (7☿, 4♀); *B. hortorum* (2☿)

### Laboratory protocols and sequence data set

We obtained new *cytochrome c oxidase subunit I* (COI) gene sequences from 12 Icelandic bumblebee specimens (Table 2). Molecular analysis (purification and PCR) was performed at the Federal Center for Integrated Arctic Research of the Russian Academy of Sciences. A total DNA was extracted from a head capsule of each dried specimen using a standard phenol-chloroform procedure (Sambrook et al. 1989). The COI gene was amplified and sequenced using primer pairs C1-J-1718 and C1-N-2329R (Simon et al. 1994). The PCR mix contained approximately 200 ng of total cell DNA, 10 pmol of each primer, 200 μmol of each dNTP, 2.5 μl of PCR buffer (with 10 × 2 mmol MgCl_2_), and 0.8 units Taq DNA polymerase (SibEnzyme Ltd.); H_2_O was added for a final volume of 25 μl. Temperature cycling was as follows: 95 °C (4 min), 40 cycles of 95 °C (45 sec), 48‒53 °C (40 sec), 72 °C (50 sec) and a final extension at 72 °C (5 min). The sequencing was carried out at the Engelhardt Institute of Molecular Biology of the Russian Academy of Sciences (Moscow) using the ABI PRISM® BigDye Terminator v. 3.1 reagent kit. Reaction products were analyzed using an automatic sequencer, ABI PRISM 3730 (Applied Biosystems). The obtained results were analyzed using BioEdit version 7.2.5 (Hall 1999). Additionally, 165 COI sequences were obtained from the NCBI GenBank and the Barcode of Life Database (BOLD) (Suppl. material 1).

**Table 2.** List of COI barcode sequences for bumblebee specimens from Iceland

Species	COI lineage	COI GenBank acc. no.	Specimen Voucher*	Locality
*Bombus jonellus*	BJ-01	MH168019	BMB35	Blanda River
	BJ-01	MH168020	BMB50	Mývatn Lake
	BJ-01	MH168022	BMB54	Mývatn Lake
	BJ-01	MH168027	BMB71	Reykjavík
	BJ-01	MH168028	BMB75	Reykjavík
	BJ-01**	MH168025	BMB66	Reykjavík
	BJ-02	MH168021	BMB52	Mývatn Lake
*B. lucorum*	BL-01	MH168017	BMB19	Blanda River
	BL-01	MH168024	BMB64	Reykjavík
	BL-01	MH168018	BMB28	Blanda River
	BL-01	MH168023	BMB63	Reykjavík
*B. hortorum*	BH-01	MH168026	BMB70	Reykjavík

*Deposited in the collection of the Russian Museum of Biodiversity Hotspots, Federal Center for Integrated Arctic Research of the Russian Academy of Sciences, Arkhangelsk, Russia. **This specimen shares a specific singleton, which differs from the other haplotype in lineage BJ-01 (497 A vs. 497 T).

### Sequence alignment and phylogeographic analyses

The alignment of COI sequences was performed using the ClustalW algorithm implemented in MEGA6 (Tamura et al. 2013). Each COI sequence of the aligned datasets was trimmed, leaving a 455-bp fragment for *Bombus jonellus*, 448-bp for *B. lucorum*, and 423-bp for *B. hortorum*. The phylogeographic analyses were performed based on a median-joining network approach using Network version 4.6.1.3 software with default settings (Bandelt et al. 1999). Genetic divergences and nucleotide substitutions were estimated in MEGA6 (Tamura et al. 2013).

## Results

Three bumblebee species were recorded in our new samples from Iceland, i.e., *Bombus jonellus*, *B. lucorum* and *B. hortorum* (Table 1). The first two species were common and widespread, while *B. hortorum* appears to be rare and has been collected from only the Reykjavík area.

**Figure 2.**
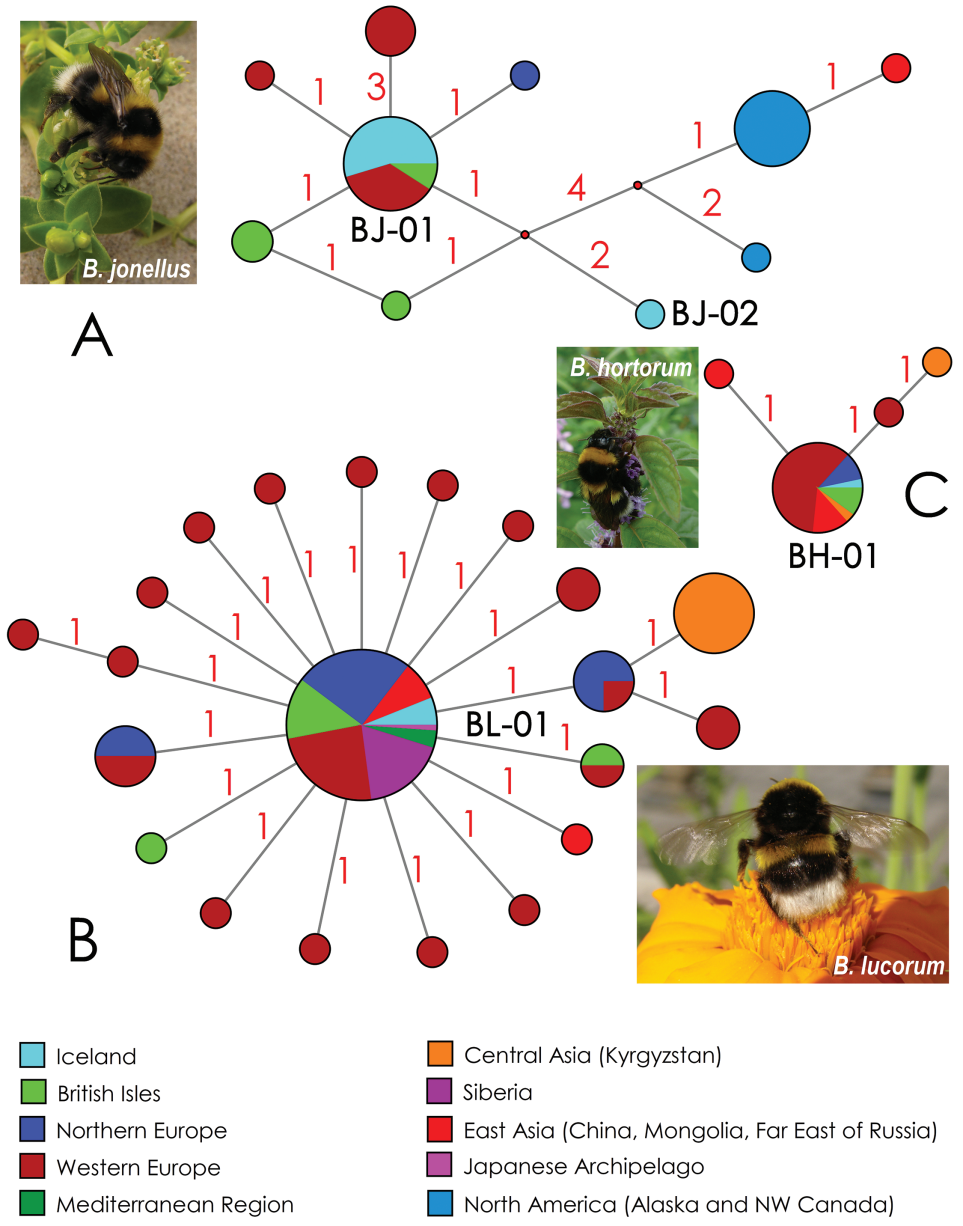
Median-joining haplotype networks of the available COI sequences of bumblebee species inhabiting Iceland. The circle size is proportional to the number of available sequences belonging to a certain haplotype. The small red dots indicate hypothetical ancestral haplotypes. Red numbers near branches indicate the number of nucleotide substitutions between haplotypes. Black codes indicate the COI lineages inhabiting Iceland. **A** *Bombus jonellus* (N = 29) **B** *B. lucorum* (N = 115) **C** *B. hortorum* (N = 33). Photographs by Yulia S. Kolosova.

**Figure 3.**
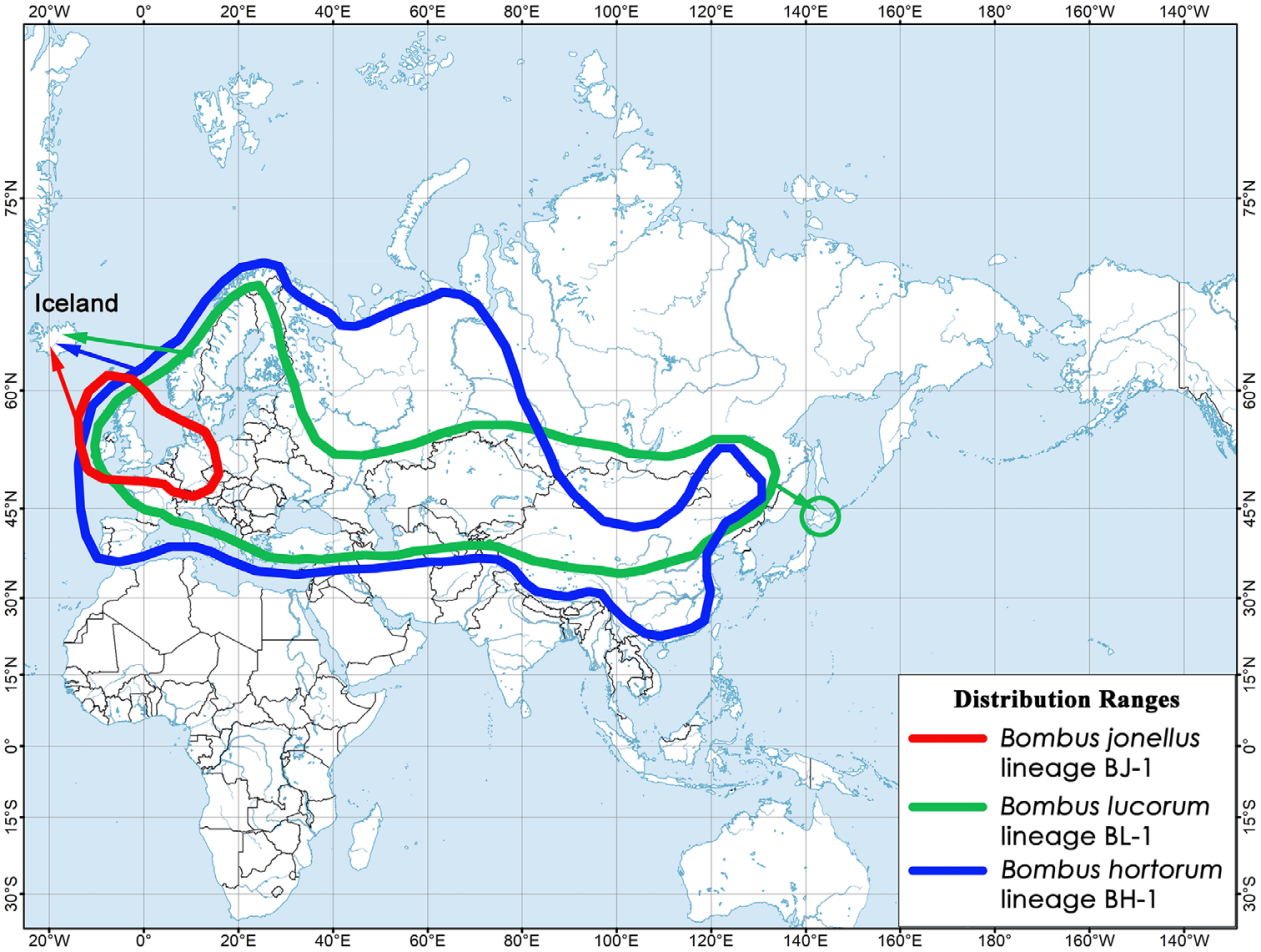
Approximate distribution ranges of the widespread ubiquistic COI lineages recorded in bumblebee populations from Iceland (see Suppl. material 1 for details). Northern range boundaries of *Bombus lucorum* and *B. hortorum* lineages are in accordance with published sources (Williams et al. 2012, Kolosova et al. 2016, Potapov and Kolosova 2016). The map was created using ESRI ArcGIS 10 software (www.esri.com/arcgis). The topographic base of the map was created with Natural Earth Free Vector and Raster Map Data (www.naturalearthdata.com).

We found that the sequenced *Bombus jonellus* specimens from Iceland share three COI haplotypes belonging to two different lineages (Fig. 2). The first lineage (BJ-01) appears to be more common in Iceland, as it was found in six sequenced specimens, one of which shares a specific singleton, with a non-synonymous substitution in pos. no. 497 (A instead of T) (Table 2). This singleton is not shown on the network illustrated in Fig. 2 because it was calculated on the basis of a short-sequence dataset (see Materials and methods). The lineage BJ-01 has also been recorded from populations on the British Isles (Ireland) and in mainland Europe (Germany) (Figs 2, 3). In summary, eleven specimens belong to this lineage (37% of the total sample of the species [*N* = 29]; see Suppl. material 1). The second *Bombus jonellus* lineage (BJ-02) appears to be rare and was found in a single specimen collected from the shore of Lake Mývatn. This lineage has not previously been recorded anywhere. It differs from the lineage BJ-01 in three non-synonymous nucleotide substitutions (218 T vs. 218 A, 284 T vs. 284 C, and 383 T vs. 383 C). The mean uncorrected COI p-distance between the lineages BJ-01 and BJ-02 is 0.5 ± 0.3%. The haplotype network of *Bombus jonellus* reveals two shallow but geographically distinct clades, i.e. the European (including Iceland) and Nearctic – Northeast Asian haplogroups (Fig. 2) that may reflect two cryptic glacial refugia.

The Icelandic *Bombus lucorum* specimens belong to a single COI lineage (BL-01) that occurs in populations from Russia, China, Mongolia, Northern Europe (Denmark, Finland, Sweden, and Latvia), Western Europe (Austria and Germany), the British Isles (Ireland and United Kingdom), and Turkey, and in an invasive population from Hokkaido, Japan (Takahashi et al. 2017) (Figs 2, 3). In general, 83 specimens belong to this lineage (72% of the total sample of the species [N = 115]; see Suppl. material 1). The star-shaped network may indicate a sudden population expansion in this species, most likely since the Last Glacial Maximum.

A single sequenced specimen of *Bombus hortorum* from Iceland also belongs to a widespread COI lineage (BH-01) that occurs in populations from China, European Russia, Northern Europe (Denmark and Norway), Western Europe (France and Germany), Southern Europe (Italy), and the British Isles (United Kingdom) (Figs 2, 3). In general, 30 specimens belong to this lineage (90% of the total sample of the species [N = 33]; see Suppl. material 1). The network with four haplotypes is too simple, but it has a rather star-like shape, most likely indicating a rapid postglacial expansion or recent human-mediated dispersal of *Bombus hortorum*.

## Taxonomy

### Bombus (Pyrobombus) jonellus

Taxon classificationAnimaliaHymenopteraApidae

Kirby, 1802

= Bombus (Pyrobombus) jonellus subborealis Richards, 1933, **syn. n.**

#### Remarks.

This subspecies is thought to be distributed in Norway and Iceland (Richards 1933, Løken 1973, Kratochwil 2016). However, our molecular data (Fig. 2) clearly indicate that this geographic race should be considered a synonym of *Bombus jonellus*. There are three other subspecies of *Bombus jonellus* from Northern Europe, i.e. *B. j. vogtii* Richards, 1933 from Shetland, *B. j. hebridensis* (Wild, 1931) from Hebrides, and *B. j. suecicus* (Friese, 1911) from Sweden (Kratochwil 2016). However, the close geographic proximity of the type localities of the named taxa raises questions about their validity and deserves further research.

## Discussion

### Origin of recent immigrants in bumblebee fauna of Iceland

The modern bumblebee fauna of Iceland with seven species is close to species-poor assemblages on boreal European islands (e.g. the Solovetsky Archipelago in Northern European Russia: Bolotov et al. 2013) but is entirely different from those on the Arctic Ocean Islands, the faunas of which are dominated by cold-adapted Arctic species (Kolosova and Potapov 2011, Potapov et al. 2017). Based on long-term collection data, Prŷs-Jones et al. (2016) have suggested that *Bombus hortorum* and *B. lucorum* invaded Iceland around the middle of the 20^th^ century (in the 1950s and in the 1970s, respectively). We discovered that the most common and widespread mtDNA lineages are found in the Icelandic populations of both species (Fig. 2). However, *Bombus hortorum* appears to have had a restricted range in southwest Iceland (Reykjavik and surrounding towns) until the present time, which aligns with the findings of Prŷs-Jones et al. (1981, 2016) and Kratochwil (2016).

We are unable to discuss the putative places of founders’ origin for Icelandic *Bombus hortorum* and *B. lucorum* populations due to extensive distribution ranges of the founding lineages, and they may have originated from the British Isles and from anywhere on the mainland (Fig. 3). Such lineages have more opportunities to invade remote island areas from a statistical perspective, e.g. via the arrival of a queen or queens hibernating in cargo or via intentional introductions (Prŷs-Jones et al. 2016). Additionally, widespread ubiquistic lineages appear to be adapted to a broad range of habitats and foraging sources, which could help them to establish permanent populations within an island environment (Bolotov et al. 2013, Bolotov 2014). We can assume that the three other bumblebee species, i.e. *Bombus hypnorum*, *B. pascuorum*, and *B. pratorum*, that have colonized Iceland in the beginning of the 21^st^ century may also share the most widespread and ubiquistic mtDNA lineages, like their predecessors, but this preliminary hypothesis has to be examined in a future study. Interestingly, an expansion of *Bombus pratorum* to Iceland in 2010 coincided with its appearance and establishment on the Faroe Islands (Madsen and Jensen 2011, Jensen and Madsen 2013, Witaliński and Jensen 2017), suggesting an intense natural dispersal event from the mainland. Kratochwil (2016) has shown that the bumblebee species turn-over in Iceland is driven primarily by global warming and the introduction of non-native species.

### Phylogeographic pattern in populations of *Bombus jonellus* and a prospective scenario of its expansion into Iceland

At first glance, a global phylogeographic pattern in *Bombus jonellus* may reflect its survival in two distant glacial refugia, i.e., in Europe and in Beringia, although no sequences of this species from Siberia are available, and may narrow the current gap between European and Nearctic haplogroups (Fig. 2). Based on our preliminary survey, we assume that the only nominative subspecies of *Bombus jonellus* is ranged in Europe because all of the European COI haplotypes of this species are quite similar and belong to a single compact haplogroup (Fig. 2).

The presence of putative unique haplotypes in Norway, the United Kingdom, and Iceland could indicate a rapid northwestern expansion of this species from glacial refugia in Southern and Central Europe in the Late Pleistocene or Early Holocene. Our data set is very limited, and it is highly likely that the unique lineage BJ-02 from Iceland can be found somewhere on the British Isles, Shetland, and Hebrides or in mainland Europe. However, our results indicate that *Bombus jonellus* may have colonized Iceland two times and that the lineage BJ-02 may reflect a more ancient, Late Pleistocene or Early Holocene founder event (e.g. from the British Isles), albeit more sampling efforts are necessary to obtain a fully resolved biogeographic model for this species. We agree with Prŷs-Jones et al. (1981, 2016) that the first expansion of *Bombus jonellus* to Iceland was most likely caused by a historical, human-mediated dispersal event. The Viking period, when large numbers of cargo ships could have supported long-distance dispersal processes in several species, e.g. the Orkney house mouse lineage (Searle et al. 2009), appears to be the most probable time of this expansion.

Indeed, our results inferred from the Icelandic bumblebees correspond well to the *tabula rasa* hypothesis. Such a phylogeographic pattern has been discovered in several other taxa, and a slowly growing body of molecular research indicates that invertebrate faunas on the North Atlantic Islands have had postglacial allochthonous origin (Pálsson et al. 2016, Bolotov et al. 2017, Vinarski et al. 2017). The Icelandic subterranean amphipods, the only known exception, were able to survive in groundwater reservoirs under glaciers during the Last Glacial Maximum (Kornobis et al. 2010). Finally, we could conclude that environmental conditions supporting the survival of freshwater and terrestrial invertebrates were lacking in Iceland during the LGM, and they may have arrived on the island after its deglaciation (pond snails: Bolotov et al. 2017, caddisflies: Pálsson et al. 2016, bumblebees: this study). This phylogeography-based conclusion is in agreement with paleogeographic modelling that suggests the existence of a continuous, thick ice sheet covering the entire island (Bingham et al. 2003, Ingólfsson et al. 2010). More interestingly, a phylogeographic pattern has recently been discovered on the Novaya Zemlya Archipelago that is thought to have served as a cryptic glacial refugium for bumblebees during the Late Pleistocene epoch (Potapov et al. 2017).

## Supplementary Material

XML Treatment for Bombus (Pyrobombus) jonellus

## Supplementary materials

Supplementary material 1 Table S1 Data type: Dataset of sequences Explanation note: List of additional COI sequences of *Bombus* species obtained from GenBank or BOLD, including taxon, accession number, specimen code, locality information and data source. This is an open access article distributed under the terms of the Creative Commons Attribution License (CC BY 4.0), which permits unrestricted use, distribution, and reproduction in any medium, provided the original author and source are credited.Grigory S. Potapov, Alexander V. Kondakov, Yulia S. Kolosova, Alena A. Tomilova, Boris Yu. Filippov, Mikhail Yu. Gofarov, Ivan N. Bolotov
