# Supplementary material for: Widespread continental mtDNA lineages prevail in the bumblebee fauna of Iceland
Source: Zookeys. 2018 Jul 16;(774):141–53. doi: 10.3897/zookeys.774.26466 (PMC6056568; doi:10.3897/zookeys.774.26466)
Supplement: Supplementary material 1 — Table S1 [file zookeys-774-141-s001.docx]

**Table S1.** List of additional *COI* sequences of *Bombus* species obtained from GenBank or BOLD, including taxon, accession number, specimen code, locality information and data source

| **Species** | **Haplotype Code** | **GenBank or BOLD acc. no.** | **Voucher no.** | **Specimen locality** |
| --- | --- | --- | --- | --- |
| *B. jonellus* | BJ-03 | KU874435 | UAM:Ento:187373 | USA: Alaska, Fairbanks, UAF Farm |
| *B. jonellus* | BJ-03 | KU874434 | UAM:Ento:187356 | USA: Alaska, Fairbanks, UAF Farm |
| *B. jonellus* | BJ-03 | KR791608 | BIOUG16746-A08 | Canada: Yukon Territory, Ivvavik National Park |
| *B. jonellus* | BJ-03 | KR783360 | BIOUG17053-C03 | Canada: Northwest Territories, Nahanni National Park |
| *B. jonellus* | BJ-03 | KR932410 | BIOUG16874-B03 | Canada: Northwest Territories, Nahanni National Park |
| *B. jonellus* | BJ-04 | AF279543 | n/a | Russia: Magadan |
| *B. jonellus* | BJ-05 | AY181113 | n/a | Norway |
| *B. jonellus* | BJ-06 | KJ839707 | BC ZSM HYM 08992 | Germany: Sachsen, Vogtland, Oelsnitz |
| *B. jonellus* | BJ-06 | KJ839685 | BC ZSM HYM 08994 | Germany: Saxony-Anhalt, Harz, Brocken |
| *B. jonellus* | BJ-07 | KJ838668 | BC ZSM HYM 01079 | Germany: Bavaria, Nuernberg |
| *B. jonellus* | BJ-01 | KJ837599 | BC ZSM HYM 01076 | Germany: Bavaria, Grafenau |
| *B. jonellus* | BJ-01 | KJ837704 | BC ZSM HYM 08993 | Germany: Saxony-Anhalt, Harz, Brocken |
| *B. jonellus* | BJ-06 | KJ837105 | BC ZSM HYM 08995 | Germany: Saxony-Anhalt, Harz, Brocken |
| *B. jonellus* | BJ-08 | JX831201 | CHU06-BEE-064 | Canada: Manitoba, Churchill, 26 km SE Churchill, Twin Lakes |
| *B. jonellus* | BJ-09 | JX830748 | CHU06-BEE-043 | Canada: Manitoba, Churchill, 4 km SE Churchill, Akudlik Marsh |
| *B. jonellus* | BJ-03 | JX830792 | CHU07-BEE-014 | Canada: Manitoba, Churchill, 26 km SE Churchill, Twin Lakes |
| *B. jonellus* | BJ-01 | JQ909704 | TCDB-T373 | Ireland: Kildare, Royal Canal, Leixlip |
| *B. jonellus* | BJ-11 | BEEEE279-16 | BMNH(E)#1730779 | UK: England, Ilkley |
| *B. jonellus* | BJ-10 | BEEEE309-16 | BMNH(E)#1730809 | UK: England, Snowden |
| *B. jonellus* | BJ-10 | BEEEE192-15 | BMNH(E)#970561 | UK: England, Oxfordshire |
| *B. jonellus* | BJ-01 | GMGRA1668-13 | BIOUG05235-B02 | Germany: Bavaria, Niederbeyrn, Bayerischer National Park |
| *B. jonellus* | BJ-01 | GMGRD3613-13 | BIOUG06436-C03 | Germany: Bavaria, Niederbeyrn, Bayerischer National Park |
| *B. hortorum* | BH-01 | KY271879 | VSU_B_4 | Russia: European part |
| *B. hortorum* | BH-01 | AY181104 | n/a | Denmark |
| *B. hortorum* | BH-01 | AY181103 | n/a | Germany |
| *B. hortorum* | BH-01 | AY181102 | n/a | France |
| *B. hortorum* | BH-02 | KP671630 | HO000TR | Turkey: Artvin |
| *B. hortorum* | BH-01 | KP671629 | HO00074 | China: Yamate |
| *B. hortorum* | BH-01 | KP671628 | HO00082 | China: Xiaodonggou |
| *B. hortorum* | BH-03 | KP671627 | HO00081 | China: Xiaodonggou |
| *B. hortorum* | BH-01 | KP671626 | HO00022 | China: Guazigou |
| *B. hortorum* | BH-01 | KJ734243 | HOOOO20 | China: Guazigou |
| *B. hortorum* | BH-01 | KJ839469 | BC ZSM HYM 15429 | Germany: Brandenburg, Mallnow |
| *B. hortorum* | BH-01 | KJ839265 | BC ZSM HYM 17914 | Germany: Baden-Wuerttemberg, Bickensohl |
| *B. hortorum* | BH-01 | KJ839185 | BC ZSM HYM 17911 | Germany: Bavaria, 2 Km NW Fischen, Ammermuendung |
| *B. hortorum* | BH-01 | KJ838942 | BC ZSM HYM 17909 | Italy: Piedmont, 2 Km W San Bernado |
| *B. hortorum* | BH-01 | KJ836944 | BC ZSM HYM 17910 | Germany: Bavaria, 8 Km E Passau |
| *B. hortorum* | BH-01 | KJ836550 | BC ZSM HYM 17907 | Germany: Baden-Wuerttemberg, Bremgarten |
| *B. hortorum* | BH-01 | HM401455 | BC ZSM HYM 01068 | Germany: Bavaria, Augsburg |
| *B. hortorum* | BH-04 | HM401454 | BC ZSM HYM 01067 | Germany: Bavaria, Herbertshausen |
| *B. hortorum* | BH-01 | GU705901 | BC ZSM HYM 01066 | Germany: Bavaria, Zwiesel |
| *B. hortorum* | BH-01 | KT074030 | BMNH(E)#970341 | UK: Greater London, London, Deptford |
| *B. hortorum* | BH-01 | GBLGC009-12 | BC ZSM HYM 12359 | Germany: Bavaria, Oberbayern, Ebersberg |
| *B. hortorum* | BH-01 | GMGMA469-14 | BIOUG16209-G10 | Germany: Rhineland-Palatinate, Kreis Ahrweiler |
| *B. hortorum* | BH-01 | GMGMA549 | BIOUG16210-F07 | Germany: Rhineland-Palatinate, Kreis Ahrweiler |
| *B. hortorum* | BH-01 | GMGMA558-14 | BIOUG16210-G04 | Germany: Rhineland-Palatinate, Kreis Ahrweiler |
| *B. hortorum* | BH-01 | GMGMC139-14 | BIOUG16356-A04 | Germany: Rhineland-Palatinate, Kreis Ahrweiler |
| *B. hortorum* | BH-01 | GMGMC223-14 | BIOUG16356-H04 | Germany: Rhineland-Palatinate, Kreis Ahrweiler |
| *B. hortorum* | BH-01 | GMGMI067-14 | BIOUG17084-F02 | Germany: Rhineland-Palatinate, Kreis Ahrweiler |
| *B. hortorum* | BH-01 | GMGMI068-14 | BIOUG17084-F03 | Germany: Rhineland-Palatinate, Kreis Ahrweiler |
| *B. hortorum* | BH-01 | GMGMJ105-14 | BIOUG17068-G12 | Germany: Rhineland-Palatinate, Kreis Ahrweiler |
| *B. hortorum* | BH-01 | AY181105 | n/a | Norway |
| *B. hortorum* | BH-01 | BEEEE289-16 | BMNH(E)#1730789 | UK: England, Wallingford |
| *B. hortorum* | BH-01 | BEEEE333-16 | BMNH(E)#1730833 | UK: England, Abergwyngregyn |
| *B. lucorum* | BL-01 | JQ843548 | 1550-608-MON | Mongolia: Hovsgol, Uur Gol |
| *B. lucorum* | BL-01 | JQ843547 | 6875C08 | Sweden |
| *B. lucorum* | BL-01 | JQ843546 | 6875C07 | Sweden |
| *B. lucorum* | BL-02 | JQ843545 | 6878E08 | UK |
| *B. lucorum* | BL-01 | JQ843544 | 6878E09 | UK |
| *B. lucorum* | BL-01 | JQ843543 | 6875C06 | Sweden |
| *B. lucorum* | BL-01 | JQ843542 | 6875C05 | Sweden |
| *B. lucorum* | BL-01 | JQ843541 | 6875C04 | Sweden |
| *B. lucorum* | BL-01 | JQ843540 | 6875C02 | Sweden |
| *B. lucorum* | BL-01 | JQ843539 | 6875C01 | Sweden |
| *B. lucorum* | BL-01 | JQ843538 | 6875E11 | China |
| *B. lucorum* | BL-01 | JQ843537 | 6878G04 | Switzerland |
| *B. lucorum* | BL-01 | JQ843536 | 6875E 07 | China |
| *B. lucorum* | BL-01 | JQ843535 | 6878H10 | Switzerland |
| *B. lucorum* | BL-01 | JQ843534 | 6878G05 | Switzerland |
| *B. lucorum* | BL-01 | JQ843533 | 6878G06 | Switzerland |
| *B. lucorum* | BL-01 | JQ843532 | 6878H07 | Switzerland |
| *B. lucorum* | BL-01 | JQ843531 | 6878G07 | Switzerland |
| *B. lucorum* | BL-01 | JQ843530 | 6878G08 | Switzerland |
| *B. lucorum* | BL-01 | JQ843529 | 6875B12 | Sweden |
| *B. lucorum* | BL-01 | JQ843528 | 6878G09 | Switzerland |
| *B. lucorum* | BL-01 | JQ843527 | 6875B11 | Sweden |
| *B. lucorum* | BL-01 | JQ843526 | 6878H06 | Switzerland |
| *B. lucorum* | BL-01 | JQ843525 | 6878G10 | Switzerland |
| *B. lucorum* | BL-01 | JQ843524 | 6873A06 | China |
| *B. lucorum* | BL-01 | JQ843523 | 6876F01 | Russia: Novosibirsk |
| *B. lucorum* | BL-01 | JQ843522 | 6876E12 | Russia: Novosibirsk |
| *B. lucorum* | BL-01 | JQ843521 | 6876F02 | Russia: Novosibirsk |
| *B. lucorum* | BL-01 | JQ843520 | 6876E10 | Russia: Novosibirsk |
| *B. lucorum* | BL-01 | JQ843519 | 6876E09 | Russia: Novosibirsk |
| *B. lucorum* | BL-01 | JQ843518 | 6876E08 | Russia: Novosibirsk |
| *B. lucorum* | BL-01 | JQ843517 | 6876F03 | Russia: Novosibirsk |
| *B. lucorum* | BL-01 | JQ843516 | 6873A07 | China |
| *B. lucorum* | BL-01 | JQ843515 | 6876F06 | Russia: Novosibirsk |
| *B. lucorum* | BL-01 | JQ843514 | 6876F07 | Russia: Novosibirsk |
| *B. lucorum* | BL-01 | JQ843513 | 6876E01 | Iceland |
| *B. lucorum* | BL-03 | JQ843512 | 1550-607-MON | Mongolia: Hovsgol, Uur Gol |
| *B. lucorum* | BL-01 | JQ843511 | 6876F08 | Russia: Novosibirsk |
| *B. lucorum* | BL-01 | JQ843510 | 6876B12 | Turkey |
| *B. lucorum* | BL-01 | JQ843509 | 6874A04 | Turkey |
| *B. lucorum* | BL-01 | JQ843508 | 6876F09 | Russia: Novosibirsk |
| *B. lucorum* | BL-01 | JQ843507 | 6876F10 | Russia: Novosibirsk |
| *B. lucorum* | BL-01 | JQ843506 | 6876F11 | Russia: Novosibirsk |
| *B. lucorum* | BL-01 | JQ843505 | 6876F12 | Russia: Novosibirsk |
| *B. lucorum* | BL-01 | JQ843504 | 6873E07 | Mongolia |
| *B. lucorum* | BL-01 | JQ843503 | 6878A05 | Latvia |
| *B. lucorum* | BL-01 | JQ843502 | 6874A10 | Turkey |
| *B. lucorum* | BL-04 | JQ843501 | 6878A06 | Latvia |
| *B. lucorum* | BL-01 | JQ843500 | 6878G11 | Switzerland |
| *B. lucorum* | BL-01 | JQ843499 | 6878A07 | Latvia |
| *B. lucorum* | BL-05 | JQ843498 | 6878G12 | Switzerland |
| *B. lucorum* | BL-01 | JQ843497 | 6874F05 | Switzerland |
| *B. lucorum* | BL-01 | JQ843496 | 6878A08 | Latvia |
| *B. lucorum* | BL-06 | JQ843494 | 6874F04 | Switzerland |
| *B. lucorum* | BL-01 | JQ843493 | 6874F03 | Switzerland |
| *B. lucorum* | BL-01 | JQ843492 | 6876F05 | Russia: Novosibirsk |
| *B. lucorum* | BL-01 | GU705910 | BC ZSM HYM 01086 | Germany: Bavaria, Miesbach |
| *B. lucorum* | BL-01 | GU705909 | BC ZSM HYM 01087 | Germany: Bavaria, Pfaffenkogel BGL |
| *B. lucorum* | BL-06 | GU705906 | BC ZSM HYM 01085 | Germany: Bavaria, Kehlheim, Siegenburg |
| *B. lucorum* | BL-07 | LN714040 | n/a | Austria: Carinthia, Hohe Tauern southern slope |
| *B. lucorum* | BL-08 | LN714039 | n/a | Austria: Carinthia, Heiligenblut |
| *B. lucorum* | BL-01 | LN714038 | n/a | Austria: Lower Austria, Lunz am See |
| *B. lucorum* | BL-05 | LN714037 | n/a | Austria: Salzburg, Tennen Mountains |
| *B. lucorum* | BL-04 | LN714036 | n/a | Austria: Lower Austria, Lunz am See |
| *B. lucorum* | BL-09 | LN714035 | n/a | Austria: Lower Austria, Raxalpe |
| *B. lucorum* | BL-10 | LN714034 | n/a | Austria: Carinthia, Hohe Tauern southern slope |
| *B. lucorum* | BL-01 | LN714033 | n/a | Austria: Carinthia, Karawanks |
| *B. lucorum* | BL-11 | LN714032 | n/a | Austria: Lower Austria, Blockheide |
| *B. lucorum* | BL-12 | LN714031 | n/a | Austria: Lower Austria, Lunz am See |
| *B. lucorum* | BL-02 | LN714030 | n/a | Austria: Vienna, Vienna Woods |
| *B. lucorum* | BL-13 | LN714029 | n/a | Austria: Carinthia, Karawanks |
| *B. lucorum* | BL-14 | LN714028 | n/a | Austria: Lower Austria, Raxalpe |
| *B. lucorum* | BL-15 | LN714027 | n/a | Austria: Salzburg, Tennen Mountains |
| *B. lucorum* | BL-16 | LN714026 | n/a | Austria: Lower Austria, Lunz am See |
| *B. lucorum* | BL-17 | LN714025 | n/a | Austria: Lower Austria, Raxalpe |
| *B. lucorum* | BL-01 | LN714024 | n/a | Austria: Vienna, Vienna Woods |
| *B. lucorum* | BL-01 | JQ692957 | 6875C03 | Sweden |
| *B. lucorum* | BL-01 | JN872605 | T690 | Ireland: Wicklow, Powerscourt |
| *B. lucorum* | BL-18 | JN872604 | T691 | Ireland: Wicklow, Clara |
| *B. lucorum* | BL-01 | JN872603 | T692 | Ireland: Kerry, Killarney |
| *B. lucorum* | BL-01 | JN872602 | T716 | Ireland: Wicklow, Powerscourt |
| *B. lucorum* | BL-01 | JN872601 | T718 | Ireland: Wicklow, Powerscourt |
| *B. lucorum* | BL-01 | JN872600 | T728 | Denmark: Jutland, Stubbergaard So |
| *B. lucorum* | BL-01 | JN872599 | T729 | Denmark: Jutland, Holstebro |
| *B. lucorum* | BL-01 | JN872598 | T730 | Denmark: Jutland, Eshoj Plantage |
| *B. lucorum* | BL-01 | JN872597 | T731 | Denmark: Jutland, Gram Storskov |
| *B. lucorum* | BL-01 | JN872596 | T767 | Denmark: Jutland, Soby |
| *B. lucorum* | BL-01 | JN872595 | T787 | Ireland: Kerry, Killarney |
| *B. lucorum* | BL-01 | JN872594 | T791 | United Kingdom: Scotland, Orkney, Harray, Netherborough |
| *B. lucorum* | BL-01 | JN872593 | T792 | UK: Scotland, Orkney, Burray, Bu Links |
| *B. lucorum* | BL-01 | JN872592 | T875 | Finland |
| *B. lucorum* | BL-01 | JN872591 | T880 | Finland |
| *B. lucorum* | BL-01 | JN872590 | T887 | Finland |
| *B. lucorum* | BL-04 | JN872589 | T904 | Finland: Naantali |
| *B. lucorum* | BL-04 | JN872588 | T909 | Finland |
| *B. lucorum* | BL-01 | LC123695 | n/a | Japan: Hokkaido |
| *B. lucorum* | BL-19 | KJ838784 | BC ZSM HYM 13414 | Germany: Baden-Wuerttemberg, Grissheim |
| *B. lucorum* | BL-01 | KX791759 | n/a | China: Inner Mongolia |
| *B. lucorum* | BL-20 | KX957832 | n/a | Kyrgyzstan |
| *B. lucorum* | BL-20 | KX957831 | n/a | Kyrgyzstan |
| *B. lucorum* | BL-20 | KX957830 | n/a | Kyrgyzstan |
| *B. lucorum* | BL-20 | KX957874 | n/a | Kyrgyzstan |
| *B. lucorum* | BL-01 | AY694095 | n/a | UK: Crail, Scotland |
| *B. lucorum* | BL-01 | AY530010 | n/a | Germany: Menz |
| *B. lucorum* | BL-01 | AY530009 | n/a | Germany: Menz |
| *B. lucorum* | BL-21 | AY181120 | n/a | Norway |
| *B. lucorum* | BL-21 | AY181119 | n/a | Denmark |
| *B. lucorum* | BL-21 | AY181118 | n/a | Switzerland |
| *B. lucorum* | BL-21 | AY181117 | n/a | Austria |
| *B. lucorum* | BL-01 | BEEEE219-15 | BMNH(E)#970587 | UK: London |
| *B. lucorum* | BL-01 | BEEEE234-15 | BMNH(E)#970600 | UK: London |

n/a – not available.
